# Difference in Pandemic-Related Experiences and Factors Associated with Sickness Absence among Nurses Working in COVID-19 and Non-COVID-19 Departments

**DOI:** 10.3390/ijerph19031093

**Published:** 2022-01-19

**Authors:** Matea Dolić, Vesna Antičević, Krešimir Dolić, Zenon Pogorelić

**Affiliations:** 1 Department of Health Studies, University of Split, 21000 Split, Croatia; vesna.anticevic@ozs.unist.hr; 2 Department of Diagnostic and Interventional Radiology, University Hospital of Split, 21000 Split, Croatia; kdolic79@gmail.com; 3 School of Medicine, University of Split, 21000 Split, Croatia; zpogorelic@gmail.com; 4 Department of Pediatric Surgery, University Hospital of Split, 21000 Split, Croatia

**Keywords:** sickness absence, coping strategies, personality traits, COVID-19, nurses

## Abstract

Background: The aim of this study is to determine the main variables associated with nurses’ sickness absence (SA) and to improve the prediction of SA based on pandemic-related experiences. The second aim is to examine the differences between COVID-19 (CoV) and non-COVID-19 (non-CoV) nurses in levels of post-traumatic stress disorder (PTSD) symptoms, personality traits, coping strategies and professional stressors experienced. Methods: This historical prospective study enrolled 1305 nurses from the University Hospital of Split, Croatia. A total of 380 subjects participated in the study, 163 non-CoV and 217 CoV subjects. Nurses’ pandemic-related experience questionnaires, Big Five Inventory (BFI), Post-traumatic Stress Disorder Checklist (PCL-5), Coping Inventory for Stressful Situations (CISS) and Occupational Stress Questionnaire, were used for evaluation. Results: Non-CoV nurses felt more fear of infection, were more socially distanced, had more PTSD symptoms and neuroticism and felt more stress due to public criticism and job requirements compared to CoV nurses; *p* < 0.001. The groups of SA users and non-SA users could be distinguished based on predictor variables in CoV and non-CoV nurses, with a correct classification of 84.8% vs. 79.1%. Conclusions: It was possible to predict the probability of using SA among nurses due to pandemic professional experience, personality traits and coping strategies.

## 1. Introduction

The World Health Organisation (WHO) declared the COVID-19 pandemic in March 2020 due to the rapid spread of coronavirus globally [1]. It poses unique challenges, both due to the impact it has on health systems and the degree of personal risk it places upon those who work in healthcare, in particular on the front line in hospitals and long-term care facilities [2,3,4]. The pandemic has caused a large increase in the workload not only because of the sheer number of patients requiring treatment for illness, but also because of the need to do more due to the absence of colleagues who tested positive for coronavirus, in isolation or self-isolation due to close contact with infection or personal serious risk factors that could adversely affect the clinical outcome in the event of virus infection. It definitely represents a new challenge in the context of sick absence (SA) in the health system, in which there was already a shortage of staff even before the pandemic [4,5,6,7]. Nurses play a crucial role in providing health care and, as such, are the most exposed in this pandemic. At the same time, the shortage of nursing personnel severely affects the quality of medical services globally. This problem has been trending in most countries around the world even before this pandemic [7]. Another global challenge is SA in healthcare due to burnout syndrome (BOS), characterized by mental, physical and emotional exhaustion and fatigue, depression, anxiety and PTSD, with increased prevalence of suicide among HCWs. The high prevalence of BOS among health care workers (HCWs) was widely reported even before this pandemic with a potentially negatively impact on the quality and safety of patient care [8,9]. In a meta-analysis of 13 included studies using the Maslach Burnout Inventory (MBI) scale, Gómez-Urquiza et al. found that around 30% of the included nurses working in intensive care showed burnout in each of the three subscales of the MBI [9]. Nurses are very susceptible to burnout due to the specific relationship between the patient and the caregiver. This relationship requires emotional involvement in which they need to deal with a variety of possible situations, including suffering, fear, aggression, or a lack of respect for their work [10]. Research and experience, to date, have shown that nurses are willing to sacrifice their own needs during the sudden natural disasters and epidemics/pandemics of infectious diseases to actively participate and make selfless contributions out of moral and professional responsibility [11]. Due to the high workload during those public emergencies, at the same time, nurses would be in a state of physical and mental stress and would feel isolated and helpless facing health threats and work pressure. Stress can also have a significant impact on nurses and their ability to perform tasks, as well as an impact on making bad professional decisions. Job performance can also be impaired by apathy, lack of concentration, anxiety and decreased motivation that can cause uncharacteristic errors that can lead to poor clinical outcome [11,12]. That is another reason why we need to use supportive coping strategies to reduce the amount of stress and prevent the onset of burnout syndrome. These parameters should be influenced by professional experience, education level and resources available in a social context, and are usually individualised [13,14]. On the other hand, nurses’ health and patient outcomes might be compromised by long-lasting and continuous stress and inefficient coping strategies. We definitely need to better understand the needs and experiences of high-risk HCWs to be able to improve psychological support by using targeted interventions until the end of this pandemic or during similar disasters [15].

The health care system (HCS) in England recorded around 73,200 (18%) more SA days among nurses and health visitors in May 2021 than in May 2019. Over that time, the number of SAs taken for mental health reasons increased by 31% [16,17]. From a business perspective, due to increased workload, SA is an expensive issue affecting service delivery and quality due to staff shortages [18]. Therefore, it is critical to identify previous SAs among nurses so that future SAs may be predicted [19].

Because the HCS globally already struggles with thousands of vacancies, it is imperative to identify the factors resulting in staff SAs and to take steps to prevent morbidity and mortality among the staff responding to the COVID-19 pandemic [19,20,21]. 

The primary outcome of this study is to investigate whether nurses who worked in the COVID-19 department (CoV nurses) and nurses who did not work in the COVID-19 department (non-CoV nurses) differed in (a) pandemic-related experiences, (b) levels of post-traumatic stress disorder symptoms, (c) personality traits, (d) coping strategies and (e) professional stressors experienced. 

Further, the secondary aim is to investigate the association between SA with pandemic/professional-related stressors and personal features (personality traits and coping strategies), as well as post-traumatic stress symptoms among nurses working at CoV and non-CoV departments, separately. 

## 2. Materials and Methods

### 2.1. Ethical Approval

The study was approved by the Ethics Committee of the University of Split, School of Medicine (Reference: 003-08/20-03/0005; date of approval 16 November 2020) and by the Ethics Committee of the University Hospital of Split (Reference: 500-03/20-01/108; date of approval 30 October 2020) in full conformance with the principles of the Declaration of Helsinki for Good Clinical Practice (GCP).

### 2.2. Participants

This historical prospective study was conducted among 1305 nurses employed at the University Hospital of Split, Croatia, in December 2020.

Among them, 250 frontline nurses were reassigned to work in the hospital COVID-19 unit treating the most severe cases of patient with COVID-19 disease (Group 1), while 1055 were working in non-COVID-19 departments treating patients who were seeking hospital care for symptoms of diseases other than COVID-19 disease (Group 2) during the first pandemic wave. The groups were formed according to the answer to the question “Did you work at a COVID-19 department during the coronavirus pandemic?”

Inclusion criteria: nurses employed at the University Hospital of Split who worked during the first wave of the COVID-19 pandemic. Exclusion criteria: long-term sickness absences, especially during the pandemic’s first wave, and incomplete forms. 

The online survey link was sent to all 1305 participants via their official corporate email. The online form contained information on the purpose of the research study, guaranteed anonymity and asked for consent to participate in the research study. Pressing the “Agree” button was considered as consent to participate in the survey. This was followed by questions about sociodemographic characteristics and sickness absence from the beginning of the pandemic, followed by the questionnaires used in this study. After completing the form, participants had to press the “Submit” button to confirm their participation. The data were automatically recorded into an Excel spreadsheet. Only participants who completed the entire online form were eligible for further processing, while incomplete forms were not registered by Google forms. We set a two-week deadline to complete the survey. Two reminder emails were sent, the first after five days and the second after ten days, with an invitation to participate in the research study. The data were collected by the co-investigators, entered into an Excel spreadsheet and were coded and double-checked by the PI (the PI was the link between the data and code list). The data were stored in a protected computer by the researcher in accordance with the corporate policies and guidelines.

**Figure 1:**
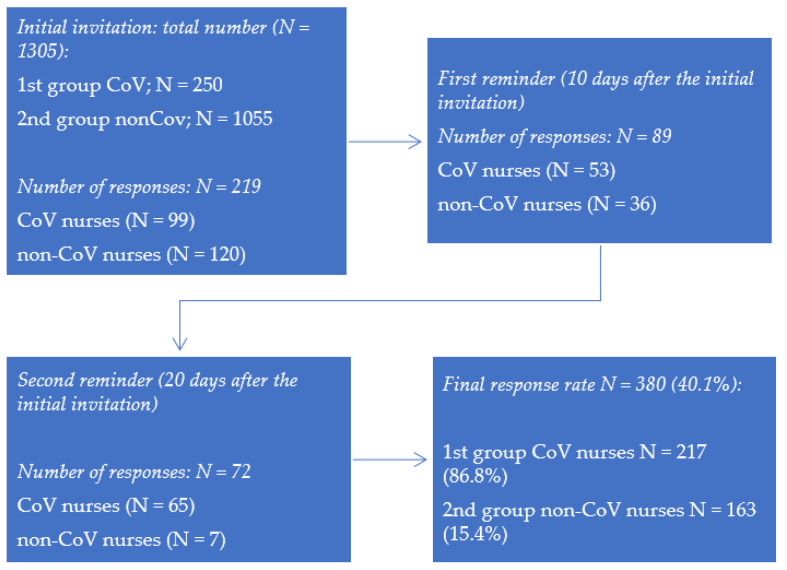
Flow chart of data collection and response rates.

The sampling procedure and response rates are shown in Figure 1.

### 2.3. Measures

#### 2.3.1. Demographic Information

For the purpose of this research study, a general information questionnaire to collect the participants’ demographic information was prepared. 

#### 2.3.2. Nurses’ Pandemic-Related Experiences Questionnaire 

The questionnaire consisted of nine statements to examine the personal experience of nurses working with COVID-19-positive patients during the first wave of the pandemic. The participants responded on a scale from 1 (“does not apply to me at all”) to 5 (“fully applies to me”). The total score of each participant was expressed as the final sum of responses to each statement. The analysis of the main components performed with the Promax rotation method disclosed the three-factor structure of the questionnaire. The first subscale, “Stigmatization and misunderstanding”, reflected feelings of stigma that nurses experienced while working with COVID-19 patients. The second subscale, “Social distancing”, described actual or planned distancing/avoidant behaviours of nurses in order to protect significant others. The third subscale, “Fear of infection”, described nurses’ fears of infecting oneself or loved ones. The Cronbach’s alpha coefficients vary between 0.81 and 0.88, indicating good internal reliabilities of all three subscales. 

#### 2.3.3. The Big Five Inventory (BFI) 

The Big Five Inventory (BFI) [22] was used to assess five major dimensions of personality, namely, extraversion, agreeableness, conscientiousness, neuroticism and openness to experience. The questionnaire consisted of 44 statements. the participants expressed their degree of agreement with each of the statements, on a scale from 1 to 5 (1—“completely disagree”; 5—“completely agree”). The score of the participants was determined by summing the estimates for the corresponding items of each dimension of the questionnaire, which allowed us to obtain the total score for the dimensions of the BFI. In spite of its brevity, the BFI does not compromise content coverage or good psychometric properties. The preliminary results of verifying the psychometric characteristics of the Croatian version of this questionnaire retained satisfactory psychometric characteristics [23]. 

#### 2.3.4. Post-Traumatic Stress Disorder Checklist (PCL-5)

PCL-5 is a 20-item questionnaire for assessing post-traumatic symptoms in the last month according to the DSM-5 criteria [24]. For the purpose of this study, the participants estimated their reactions to COVID-19 exposure. They were asked to indicate the number on a scale from 0 (“not at all”) to 4 (“extremely”) referring to the worst event according to his/her own experience. The overall result ranges from 0 to 80, where a PCL-5 cut-off score between 31 and 33 is indicative of probable PTSD, while a score of 33 or higher is used to indicate a high level of PTSD. Previous research has found good psychometric properties and reliability of the PCL-5 [25].

#### 2.3.5. Coping Inventory for Stressful Situations (CISS)

For the measurement of coping with stressful situations, the Croatian version of the Endler and Parkers’ CISS [26] was used. The CISS consists of 48 items divided in 3 subscales (coping strategies) of 16 items scored from 1 (“not at all”) to 5 (“always”), with a higher score indicating more frequent use of certain coping strategies (problem-oriented coping, emotion-oriented coping and avoidant coping). The possible range of responses on each scale can vary from 16 to 80. The internal consistency Cronbach’s alpha in the Croatian version of the scale are, starting from above, 0.80, 0.82 and 0.75.

#### 2.3.6. Occupational Stress Questionnaire

A questionnaire on stressors in the workplace of hospital health workers was made based on the standardized Occupational Stress Questionnaire [27] and preliminary research. The respondents were offered 37 stressors at work related to work organization, shift work, professional advancement, education, professional requirements, interpersonal communication and communication of healthcare professionals with patients and the fear of danger and health hazards. The subjects rated their responses on a Likert scale with grades from 1 = “not stressful at all” to 5 = “extremely stressful”. The factor analysis extracted six factors of relatively high reliability of the type of internal consistency (all Cronbach’s α values greater than 0.7), i.e., workplace organization and financial issues; public criticism; dangers and harms at work; conflicts and communication at work; shift work; and professional and intellectual requirements. 

Prior to the online test, all participants gave their informed consent regarding the data they submitted. They completed the questionnaire, which lasted approximately 20 min, on their own. The data obtained based on the nurses’ responses to the scales of experience associated with the pandemic were used. Study participation was voluntary and completely anonymous and all who approached the survey answered all questions.

### 2.4. Strength of the Study

The data in Table 1 show that the expected minimum number of subjects for a test strength of 0.8 and 95% confidence interval was 2 × 162 (324) subjects in total for each observed group (dichotomous endpoint, two-independent sample study). A total of 380 subjects participated in the study, with non-CoV N = 163 and CoV N = 217 subjects.

### 2.5. Statistical Analysis

The data were recorded, sorted and prepared for analysis using the SPSS version 26.0 software package (IBM Corp., Armonk, NY, USA). The characteristics of the groups were described by descriptive parameters of frequency and percentages, as well as means and standard deviations. A t-test was used to examine differences between CoV and non-CoV nurses who worked in departments treating patients with SARS-CoV-2 during the first wave of the pandemic. Additionally, the differences between nurses who used sick leave and those who did not were also established using independent t-tests. Finally, for the purpose of identifying variables which separated nurses who used sick leave or not based on personality features and pandemic experiences, a discriminant analysis was used. The significance threshold was set at 5%.

## 3. Results

### 3.1. Differences in Pandemic Experiences, Psychological Characteristics and Psychological Symptoms

Table 2 shows that non-CoV nurses significantly felt more fear of infection, were more socially distanced, had more PTSD symptoms and neuroticism and felt more stress due to public criticism and job requirements than CoV nurses. On the other hand, avoidance strategies were more used by CoV nurses.

### 3.2. Differences in Pandemic Experiences, Psychological Characteristics and Psychological Symptoms Regarding Use of Sick Leave

Table 3 shows the characteristics of CoV nurses with respect to the use of sick leave during the pandemic. Nurses who used SA had a more pronounced fear of SARS-CoV-2 virus infection and made less use of a problem-oriented coping strategy. According to personality traits, they were less open to experiences than nurses who did not use sick leave. 

Further, Table 3 also shows the differences between non-CoV nurses with respect to whether they did or did not use sick leave. The results show that nurses who used sick leave during the first wave of the pandemic were more afraid of infection, had more PTSD symptoms and felt more stigmatized and misunderstood than nurses who worked all the time. Regarding their personality, they showed less pronounced tendency towards altruism and friendship, less conscientiousness, were less open minded and expressed more neuroticism. Further, they used less effective stress management strategies such as problem oriented coping. Finally, they had greater sensitivity to professional stressors such as organizational problems in the workplace, public criticism, conflicts and communication problems, and professional demands during the pandemic. 

In order to identify variables in nurses who used sick leave or not based on pandemic-related experiences, levels of PTSD symptoms, personality traits, coping strategies and experiencing professional stressors among CoV and non-CoV nurses, a discriminant analysis was implemented. Nurses’ experiences, personality traits, PTSD symptoms, coping strategies and professional stressors were used as independent variables, while using sick leave was treated as an outcome. 

### 3.3. Canonical Correlation Coefficients and Eigenvalues

Both canonical discriminant functions (working at CoV and non-CoV departments separately) were statistically significant (*p* < 0.001) (Table 4), indicating that the groups of sick leave users and non-sick leave users could be distinguished based on independent variables in CoV and non-CoV nurses.

Standardized beta coefficients were given for each variable in the discriminant (canonical) function showing the variable’s unique contribution to the discrimination between groups (Table 4). It is evident that the greatest contribution for CoV nurses departments had problem-oriented coping, openness, public criticism, fear of infection and organizational problems. Regarding non-CoV nurses, the greatest contribution was provided by neuroticism, stigmatization and misunderstanding, organizational problems, social distancing and fear of infection.

In other words, if CoV nurses preferred a problem-oriented approach in coping with stress, were more open to life experiences and less sensitive to criticism and organizational problems in their workplace and had less fear of infection, the possibility of using SA was less likely. On the other hand, if non- CoV nurses scored lower on neuroticism, experienced less stigmatization during the pandemic, practiced less social distancing from close ones, had less fear of infection by SARS-CoV-2 and reported less organizational problems, they probably used sick leave less frequently.

Table 5 shows that, due to independent variables, 49.1% were correctly classified in the group of CoV nurses who used sick leave vs. 96.3% not using sick leave. The absolute correct classification was 84.8%. Further, 62.2% non-CoV nurses were correctly classified in the group who used sick leave vs. 87.7% not using sick leave. The total percentage of correct classification among non-CoV nurses was 79.1%.

In other words, it was possible to classify nurses according to the possibility of using sick leave regarding pandemic professional experience, personality traits and coping strategies and this classification was much more accurate than random guessing. 

## 4. Discussion

Health care systems around the world have borne a heavy burden due to the rapid spread of COVID-19 disease [2]. Particular pressure was put on the medical staff on the front line, especially among nurses who were at greater risk of infection [4,28,29]. During the pandemic, they were more stressed because they faced a higher workload and intensity of their work, as well as being forced to implement new protocols at the same time. The results of our study showed that non-CoV nurses felt more fear of infection and were more socially distanced, had more PTSD symptoms and neuroticism and felt more stress due to public criticism and job requirements than CoV nurses. Our findings are in line with the results of a recently published study which showed that vicarious traumatization scores for front-line nurses, including scores for physiological and psychological responses, were significantly lower than those of non-front-line nurses (*p* < 0.001) [30].

Studies conducted in China [31] and in Croatia [32] have more often reported an increase in job satisfaction among employees involved in the direct care of COVID-19 patients, which is in line with our results mentioned in the previous paragraph. This may be a consequence of the public recognition of CoV nurses in relation to nurses who did not work with COVID-19 positive patients and, as such, remained under the public radar, often caring for acute patients and life-threatening patients. In addition, due to the redistribution of part of the nurses to the COVID-19 hospital, there was a lack of nurses and they could not use their vacations and, in public and even in hospital circles, they were seen as spared [32]. 

Our findings indicate a much higher response of CoV nurses than non-CoV nurses (86.8% vs. 15.4%), which indicates a greater motivation of CoV nurses to investigate factors that contribute to the psychological adjustment of nurses to the working conditions during the pandemic. It is possible that closer (physical and emotional) contact with infected patients reflects the desire of CoV nurses to find more efficient ways to adapt to these new circumstances as well as to improving care for infected patients. In addition, this finding may reflect different coping strategies of nurses in the two groups; CoV nurses tended to actively seek ways to address problems, while non-CoV nurses were more likely to use less effective strategies, such as avoiding or using SA during crises. On the other hand, this finding prevents the possibility of generalizing the findings due to the large difference in the response of nurses in both groups.

Mental health research since the beginning of the COVID-19 pandemic in the Republic of Croatia has consistently indicated the existence of mental disorders in health professionals and the types of difficulties identified have been very similar to global trends [33,34]. Based on the findings to date, risk and protective factors that contribute to the mental health outcomes of health professionals during the COVID-19 pandemic have been identified [35,36]. Although multinational studies conducted during the COVID-19 pandemic have been largely based on online research using appropriate samples and various self-assessment measurement instruments, the results consistently point to the negative impact of the COVID-19 pandemic on the psychological well-being of the general population and health workers in particular [36,37]. During the COVID-19 pandemic, health workers have adapted, innovated and accelerated work to meet the needs of patients and the community, resulting in their congestion and a significant extension of time spent at work [38]. As a result, they have had higher levels of anxiety, depression, PTSD and burnout since the beginning of the pandemic [35]. Further, the mental needs of health professionals may change over time, depending on the circumstances of work and life generally. In the early phase of such crisis situations, HCWs try to give more priority to basic human needs such as physical safety and rest. On the other hand, at its peak, they are more focused on work and support of colleagues [39]. Recently published studies on mental health outcomes among health care workers during pandemics, including Severe Acute Respiratory Syndrome Coronavirus-2 (SARS), Middle East Respiratory Syndrome (MERS), Ebola and COVID-19, as well as burn out syndrome, suggested that healthcare workers exposed to virus-related work are 1.7 times more likely to develop psychological distress and PTSD than non-exposed workers [40,41]. Moreover, even two years after the end of the SARS pandemic, 30% of health professionals with high levels of exposure to SARS patients continued to report high levels of emotional exhaustion. Compared to estimates of previous pandemics from 2002 to 2020, it was found that, from May 2019 to March 2021, the COVID-19 pandemic caused similar levels of anxiety and even exceeded the rates of depression and PTSD in health workers in relation to all past pandemics [28,42,43,44]. This is in contrast with our results, which showed more fear, stress and PTSD symptoms among non-CoV nurses. This can be explained by the fact that, during the first wave of the pandemic, there was lack of personal protective equipment, especially in non-COVID-19 departments, which could have affected the nurses’ mental health [44]. In non-COVID-19 departments, nurses used only medical masks without protective visors, overalls and other protective equipment to work with COVID-19 patients; thus, they were considered exposed to possible contamination of asymptomatic COVID-19 patients [32]. Further, in a cross-sectional study, Arnetz et al. found that the lack of protective equipment was the worst factor impacting the mental health of HCWs, especially nurses who reported more symptoms of depression, anxiety and PTSD [45].

Generally, SA is an area of concern in nursing globally due to lack of staff, even more now during the pandemic [18]. The shortage of health staff has proven to be a major indicator for SA among front-line staff as well as fear of the disease, stress, anxiety and stigmatization [46,47,48,49]. Those SA predictors definitely differ from the pre-COVID-19 ones, such as satisfaction, commitment and leadership style [7,8]. In England, compared to the time before the pandemic, the number of full-time equivalent (FTE) days lost for mental health reasons has increased by 31.4% and days lost due to chest and respiratory problems have increased by 52.5% as well as for headaches or migraine, by 51.9% [16,50]. The most common reason for staff sickness remains anxiety, stress or depression with negative implications for both the employee and the employer. Future sick leaves were clearly associated with previously prolonged SA [51]. Thus, it is critical to identify the antecedents of SA among nurse staff. For instance, Roelen et al. tried to examine SA among HCWs and found that SA episodes in the past year predicted approximately 25% of future prolonged SA and 30% within two years [52]. One or multiple personal and occupational factors increase the risk of future SA [7]. Personal demographic variables include age and work experience, job role/duties, history of sick leaves, mild aches and personality traits, while occupational factors include working environment (e.g., hospital or long-term care facilities), shift work and unplanned shifts, the organization’s safety culture and job support among employees and management. Our results showed that nurses who used SA during the first wave of pandemic, just as non-CoV nurses, felt more fear of infection and had more PTSD symptoms but also were more stigmatized and misunderstood. Further, regarding their personality, they showed less pronounced tendency towards altruism and friendship, less conscientiousness, were less open minded and expressed more neuroticism. They also used less effective stress management strategies such as problem-oriented coping. Accordingly, employers should definitely keep records regarding SA to be able to better and timely support their staff and to reduce the risk of future sick leaves. The measures that can be taken include providing and updating knowledge about COVID-19, offering psychological support, strengthening training on professionalism and reducing the number of stressors [53,54].

In line with that, due to independent variables, our analysis showed that 49.1% of nurses were correctly classified in the group of CoV nurses who used sick leave vs. 96.3% not using sick leave. The absolute correct classification was 84.8%. Further, 62.2% of non-CoV nurses were correctly classified in the group who used sick leave vs. 87.7% not using sick leave (Table 5). The total percentage of correct classifications among non-CoV nurses was 79.1%.

### Limitations

There are few limitations of this study. First, our respondents were from only one hospital with a considerably low response rate from non-CoV nurses; therefore, the generalisation of our results has yet to be verified in larger multicentric studies. Secondly, we are also aware of the disadvantages of self-administered questionnaires which may limit the depth of the nurses’ experiences. Future research should increase the response of non-CoV nurses using a different sampling methodology (e.g., send more research reminders). It might be possible to get a better understanding of the COVID-19 impact on clinical practice by interviews with nurses or adding open-ended questions. In addition, in future studies, the follow-up on the short-term and long-term psychological impacts of epidemics need to be investigated.

## 5. Conclusions

Our non-CoV nurses experienced significantly more fear of infection and were more socially distanced, had more PTSD symptoms and were more stressed by public criticism and professional job requirements than CoV nurses during the first wave of the COVID-19 pandemic. We found that it was possible to classify nurses according to the possibility of using sick leave regarding pandemic professional experience, personality traits and coping strategies with great accuracy. Hospital management and nurse leaders need to be aware of the importance of psychological support and counselling during this pandemic to reduce their intention to take sick leave and prevent burnout, thus ensuring the sustainability of health services globally.

## Figures and Tables

**Table 1:** Display of study strength–sample size.

Group	Sample Size
Group 1	162
Group 2	162
Total	324
Group incidence 1	15%
Group incidence 2	85%
Alpha	0.05
Beta	0.2
Strength	0.8

**Table 2:** Differences in pandemic experiences, psychological characteristics and psychological symptoms between nurses who worked in COVID-19 and non-COVID-19 departments.

Variables		N	M	SD	t	*p*
Nurses’ Experiences						
Stigmatization and misunderstanding	CoV nurses	217	3.75	0.881	1.32	0.187
	Non-CoV nurses	163	3.62	1.02		
Socially distanced	CoV nurses	217	3.14	0.88	−2.24	0.026
	Non-CoV nurses	163	3.34	0.86		
Fear of infection	CoV nurses	217	3.81	0.96	−4.63	<0.001
	Non-CoV nurses	163	4.24	0.86		
Personality traits						
Extraversion	CoV nurses	217	3.769	0.572	1.32	0.186
	Non-CoV nurses	163	3.692	0.546		
Comfort	CoV nurses	217	4.070	0.478	−0.80	0.426
	Non-CoV nurses	163	4.113	0.539		
Conscientiousness	CoV nurses	217	4.349	0.516	0.99	0.325
	Non-CoV nurses	163	4.298	0.488		
Neuroticism	CoV nurses	217	2.19	0.631	−2.44	0.015
	Non-CoV nurses	163	2.38	0.79		
Openness	CoV nurses	217	3.524	0.524	0.53	0.594
	Non-CoV nurses	163	3.497	0.438		
PTSD symptoms						
PCL-5 in total	CoV nurses	217	22.216	15.242	−3.71	<0.001
	Non-CoV nurses	163	28.38	16.536		
Coping strategies						
Problem-oriented coping strategy	CoV nurses	217	3.910	0.529	0.81	0.42
	Non-CoV nurses	163	3.859	0.648		
Emotions-oriented coping strategy	CoV nurses	217	2.774	0.735	−1.64	0.10
	Non-CoV nurses	163	2.907	0.814		
Avoidance	CoV nurses	217	3.54	0.660	4.53	<0.001
	Non-CoV nurses	163	3.242	0.598		
Professional stressors						
Workplace organization and financial issues	CoV nurses	217	3.734	0.865	−0.70	0.483
	Non-CoV nurses	163	3.796	0.833		
Public criticism	CoV nurses	217	3.268	1.097	−2.37	0.018
	Non-CoV nurses	163	3.53	1.015		
Dangers and harms at work	CoV nurses	217	2.960	1.034	−1.15	0.251
	Non-CoV nurses	163	3.080	0.996		
Conflicts and communication at work	CoV nurses	217	3.260	0.968	−0.43	0.667
	Non-CoV nurses	163	3.309	1.164		
Shift work	CoV nurses	217	3.588	0.972	−0.53	0.600
	No	163	3.650	1.229		
Professional and intellectual requirements	CoV nurses	217	3.227	0.920	−2.36	0.019
	No	163	3.47	1.044		

**Table 3:** Differences in pandemic experiences, psychological characteristics and psychological symptoms regarding use of sick leave separately for nurses who worked and did not work in COVID-19 departments.

Variable		Sick Leave *	N	Mean	SD	t	*p*
Stigmatization and misunderstanding	Non-CoV nurses	Yes	57	3.964	0.740		
		No	106	3.436	1.107	3.631	<0.001
	CoV nurses	Yes	53	3.712	0.866		
		No	164	3.766	0.887	−0.396	0.693
Socially distanced	Non-CoV nurses	Yes	57	3.438	0.769	0.101	
		No	106	3.292	0.898	0.877	0.28
	CoV nurses	Yes	53	3.094	1.015		
		No	164	3.158	0.830	−0.417	0.678
Fear of infection	Non-CoV nurses	Yes	57	4.570	0.467	0.061	
		No	106	4.066	0.961	0.933	<0.001
	CoV nurses	Yes	53	4.198	0.769		
		No	164	3.682	0.989	3.940	<0.001
Extraversion	Non-CoV nurses	Yes	57	3.618	0.558	0.740	
		No	106	3.732	0.538	0.052	0.21
	CoV nurses	Yes	53	3.792	0.475		
		No	164	3.761	0.601	0.385	0.701
Comfort	Non-CoV nurses	Yes	57	4.003	0.486	0.064	
		No	106	4.171	0.559	0.054	0.48
	CoV nurses	Yes	53	4.100	0.507		
		No	164	4.061	0.470	0.503	0.616
Conscientiousness	Non-CoV nurses	Yes	57	4.113	0.474	0.062	
		No	106	4.39	0.47	0.05	<0.001
	CoV nurses	Yes	53	4.304	0.537		
		No	164	4.364	0.509	−0.720	0.473
Neuroticism	Non-CoV nurses	Yes	57	2.736	0.804	0.106	
		No	106	2.175	0.722	0.070	<0.001
	CoV nurses	Yes	53	2.121	0.705		
		No	164	2.208	0.606	−0.806	0.423
Openness	Non-CoV nurses	Yes	57	3.345	0.402	0.053	
		No	106	3.579	0.437	0.042	<0.001
	CoV nurses	Yes	53	3.243	0.577		
		No	164	3.614	0.473	−4.240	<0.001
PCL-5 in total	Non-CoV nurses	Yes	57	32.789	14.641	1.939	
		No	106	26.009	17.068	1.657	0.01
	CoV nurses	Yes	53	19.377	18.337		
		No	164	23.134	14.039	−1.368	0.176
Problem-oriented coping strategy	Non-CoV nurses	Yes	57	3.577	0.642	0.085	
		No	106	4.011	0.601	0.058	<0.001
	CoV nurses	Yes	53	3.63	0.58		
		No	164	4.000	0.481	−4.188	<0.001
Emotions-oriented coping strategy	Non-CoV nurses	Yes	57	3.040	0.723	0.958	
		No	106	2.836	0.853	0.082	0.11
	CoV nurses	Yes	53	2.668	0.772		
		No	164	2.809	0.722	−1.169	0.246
Avoidance	Non-CoV nurses	Yes	57	3.060	0.506	0.067	
		No	106	3.340	0.623	0.060	<0.001
	CoV nurses	Yes	53	3.497	0.520		
		No	164	3.548	0.700	−0.568	0.571
Workplace organization and financial issues	Non-CoV nurses	Yes	57	4.033	0.728	0.096	
		No	106	3.669	0.861	0.083	0.01
	CoV nurses	Yes	53	3.838	0.678		
		No	164	3.701	0.9165	1.170	0.244
Public criticism	Non-CoV nurses	Yes	57	3.918	0.862	0.114	
		No	106	3.316	1.033	0.100	<0.001
	CoV nurses	Yes	53	3.154	0.912		
		No	164	3.304	1.151	−0.978	0.330
Dangers and harms at work	Non-CoV nurses	Yes	57	3.242	0.988	0.130	
		No	106	2.993	0.994	0.965	0.13
	CoV nurses	Yes	53	3.037	1.034		
		No	164	2.935	1.036	0.629	0.531
Conflicts and communication at work	Non-CoV nurses	Yes	57	3.554	1.082	0.143	
		No	106	3.177	1.190	0.115	0.04
	CoV nurses	Yes	53	3.252	0.878		
		No	164	3.263	0.997	−0.074	0.941
Shift work	Non-CoV nurses	Yes	57	3.463	1.321	0.175	
		No	106	3.750	1.171	0.113	0.17
	CoV nurses	Yes	53	3.645	1.032		
		No	164	3.570	0.954	0.465	0.643
Professional and intellectual requirements	Non-CoV nurses	Yes	57	3.886	0.985	0.130	
		No	106	3.245	1.010	0.098	<0.001
	CoV nurses	Yes	53	3.367	0.824		
		No	164	3.181	0.946	1.379	0.171

* Using sick leave after June 2020.

**Table 4:** Standardized canonical discriminant function coefficients.

	CoV Nurses	Non-CoV Nurses
	Function	Function
Stigmatization and misunderstanding	−0.007	0.557
Socially distanced	0.232	−0.471
Fear of infection	−0.540	0.446
Extraversion	−0.083	0.160
Comfort	−0.116	0.277
Conscientiousness	−0.023	−0.139
Neuroticism	0.410	0.670
Openness	0.561	0.670
PCL-5 total	−0.080	−0.223
Problem-oriented coping strategy	0.646	−0.203
Emotions-oriented coping strategy	0.014	−0.495
Avoidance	−0.283	−0.102
Workplace organization and financial issues	−0.365	−0.517
Public criticism	0.546	0.984
Dangers and harms at work	−0.080	0.272
Conflicts and communication at work	−0.185	−0.144
Shift work	0.245	−0.720
Professional and intellectual requirements	−0.298	0.294

**Table 5:** Number and percentage of correct identification of nurses who used sick leave.

		Using Sick Leave after June 2020	Predicted Group Membership		Total
			YES	NO	
CoV nurses	Count	Yes	26	27	53
		No	6	158	164
	%	Yes	49.1	50.9	100
		No	3.7	96.3	100
Non-CoV nurses	Count	Yes	36	21	57
		No	13	93	106
	%	Yes	63.2	36.8	100
		No	12.3	87.7	100

## Data Availability

The data presented in this study are available upon request of the respective author. Due to the protection of personal data, the data are not publicly available.
